# Association between new onset type 1 diabetes and real-world antibiotics and neonicotinoids’ exposure-related gut microbiota perturbation

**DOI:** 10.1007/s12519-022-00589-3

**Published:** 2022-07-29

**Authors:** Zhen-Ran Xu, Xiao-Xiao Yuan, Rui-Min Chen, Hai-Yan Wei, Lin-Qi Chen, Hong-Wei Du, Gui-Mei Li, Yu Yang, Xiao-Juan Chen, Xin Fang, Fei-Hong Luo

**Affiliations:** 1 Department of Pediatric Endocrinology and Inherited Metabolic Diseases, National Children’s Medical Center, Children’s Hospital of Fudan University, 399 Wan Yuan Road, Shanghai, 201102 China; 2 Fuzhou Children’s Hospitai of Fujian Medical University, Fuzhou, China; 3 Department of Endocrinology and Inherited Metabolic, Children’s Hospital Affiliated to Zhengzhou University, Henan Children’s Hospital, Zhengzhou Children’s Hospital, Zhengzhou, China; 4 Children’s Hospital of Soochow University, Suzhou, China; 5 The First Hospital of Jilin University, Changchun, Jilin China; 6 Department of Pediatric Endocrinology, Shandong Provincial Hospital Affiliated to Shandong First Medical University, Jinan, China; 7 The Affiliated Children’s Hospital of Nanchang University, Nanchang, China; 8 Department of Endocrinology, Genetics and Metabolism, The Children’s Hospital of Shanxi Province, Taiyuan, China; 9 Fujian Medical University Union Hospital, Fuzhou, China

**Keywords:** Antibiotics, Gut microbiota, Neonicotinoids, Type 1 diabetes mellitus

## Abstract

**Background:**

The real-world exposure levels of non-therapeutic antibiotics and neonicotinoids in type 1 diabetes (T1D) children and their associations as environmental triggers through gut microbiota shifts remained unknown. We thus investigated the antibiotics and neonicotinoids’ exposure levels and their associations with gut microbiota in pediatric T1D.

**Methods:**

Fifty-one newly onset T1D children along with 67 age-matched healthy controls were recruited. Urine concentrations of 28 antibiotics and 12 neonicotinoids were measured by mass spectrometry. Children were grouped according to the kinds of antibiotics’ and neonicotinoids’ exposures, respectively. The 16S rRNA of fecal gut microbiota was sequenced, and the correlation with urine antibiotics and neonicotinoids’ concentrations was analyzed.

**Results:**

The overall detection rates of antibiotics were 72.5% and 61.2% among T1D and healthy children, whereas the neonicotinoids detection rates were 70.6% and 52.2% (*P* = 0.044). Children exposed to one kind of antibiotic or two or more kinds of neonicotinoids had higher risk of T1D, with the odd ratios of 2.579 and 3.911. Furthermore, co-exposure to antibiotics and neonicotinoids was associated with T1D, with the odd ratio of 4.924. Antibiotics or neonicotinoids exposure did not affect overall richness and diversity of gut microbiota. However, children who were exposed to neither antibiotics nor neonicotinoids had higher abundance of Lachnospiraceae than children who were exposed to antibiotics and neonicotinoids alone or together.

**Conclusion:**

High antibiotics and neonicotinoids exposures were found in T1D children, and they were associated with changes in gut microbiota featured with lower abundance of butyrate-producing genera, which might increase the risk of T1D.

**Supplementary Information:**

The online version contains supplementary material available at 10.1007/s12519-022-00589-3.

## Introduction

Type 1 diabetes (T1D) is a chronic organ-specific autoimmune disease characterized by β-cell-targeted autoimmune processes and insulin deficiency [1]. It is estimated that there are over 1.11 million T1D children < 19 years old worldwide, although there are ethnic and regional differences in these estimates [2]. A ~ 2% to ~ 3% or even higher global annual incidence increasing rate and the rising proportion of cases defined by low human leukocyte antigen (HLA) risks indicate the growing effects of environmental factors [3], including infections, gut microbiota, and nutrition, on the development of T1D in addition to genetic susceptibility [1, 4, 5]. Despite recent advancements in knowledge concerning associations between gut microbiota and T1D development, much remains unknown regarding the specific role of gut microbiota in T1D [6]. T1D patients have unique gut microbiota, but lack uniformity. Some studies found a lower α diversity, high Bacteroidetes:Firmicutes ratio and low abundance of butyrate-producing species among T1D patients [4]. These characteristics might affect intestinal permeability and molecular mimicry and thereby modulate the innate and adaptive immune systems to ultimately lead to islet autoimmunity [6].

Substance intake via food, such as additives, residual pesticides, and veterinary antibiotics, can affect gut microbiota. A previous study identified perturbation of gut microbiota by antibiotics, revealing a shift in the Bacteroidetes: Firmicutes ratio due to the aminoglycoside streptomycin [7]. Use of antibiotics not only exerts short-term effects on gut microbiota but also results in prolonged pathogen susceptibility. Another study found that early life antibiotics exposure increased the risk of developing immune and metabolic diseases [8, 9]. Pulsed therapeutic antibiotics administered early in life perturbed gut microbiota and its metabolic capacities, leading to changes in T-cell populations and higher T1D incidence in mice [10], though there was no evidence that the occurrence of T1D was related to antibiotics exposures among children [4]. Moreover, oral administration of a prophylactic antibiotic mixture decreased the abundance of butyrate-producing microbes [11], with this possibly related to T1D onset.

Neonicotinoids are a class of widely used pesticides, chronic exposure to which is reportedly related to heart defects, autism spectrum disorders, and diseases of the nervous system [12]. A recent study showed that neonicotinoids affected the gut microbiota of animals, revealing that imidacloprid disrupted the balance between gut microbiota and gut–barrier function [13, 14]. However, its effect on human gut microbiota remains unclear.

Currently, antibiotics and neonicotinoids are widely used in modern agriculture and animal husbandry, and children can be exposed to antibiotics and neonicotinoids through food, water, and even environmental exposure. However, their effects on children T1D in the real world via the potential gut microbiota changes are still unknown. We hypothesized that the structure of gut microbiota in children might differ depending on their level of exposure to antibiotics and neonicotinoids, which could potentially lead to differences in the risk of T1D. In the present study, we used urinary antibiotics and neonicotinoids levels to infer their exposure levels. We analyzed the gut microbiota of children with different urine antibiotics and neonicotinoids concentrations to determine associations between differences in gut microbiota and T1D onset.

## Methods

### Study population

**Fig. 1:**
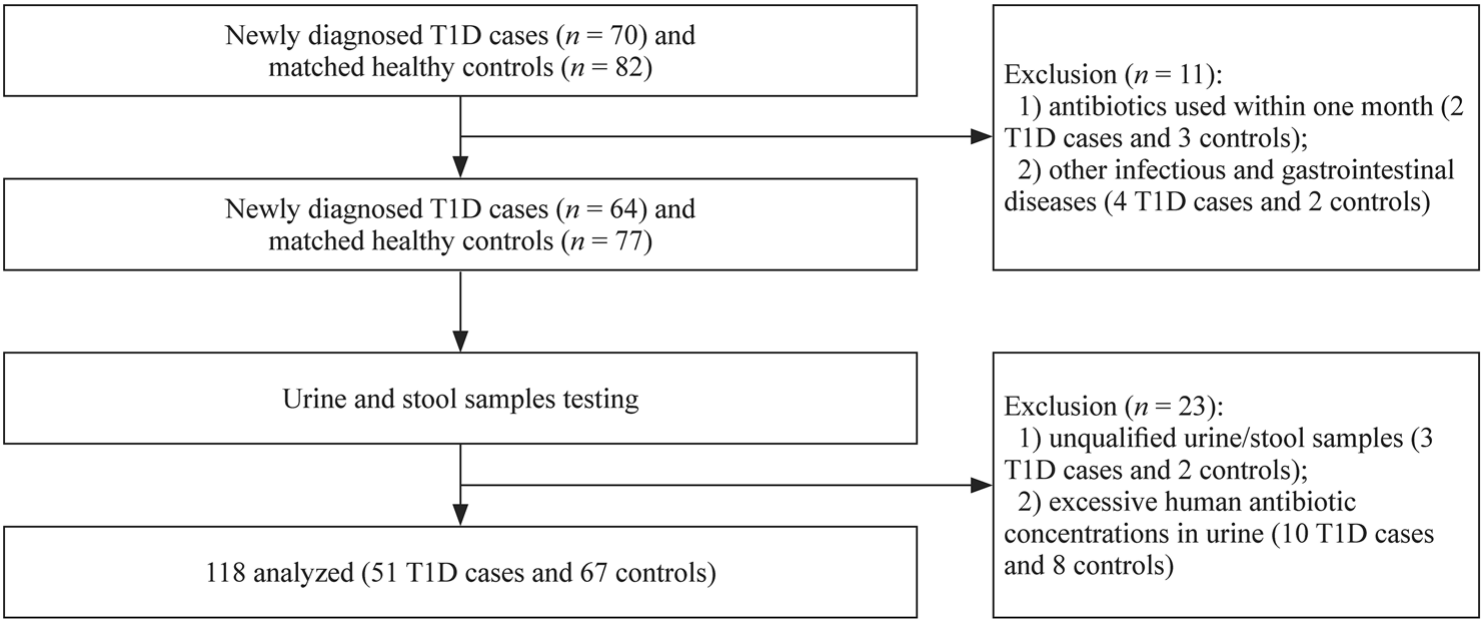
Flowchart of the inclusion and exclusion of the control and the type 1 diabetes (T1D) groups

This multi-center cross-sectional study collected samples from eight cities in different regions of China, including Fuzhou, Nanchang, Shanghai, Suzhou, Jinan, Taiyuan, Changchun, and Zhengzhou. A total of 70 newly diagnosed T1D patients < 18 years old with disease duration < 1 month were included in this study during the study period from January 2019 to March 2020. The diagnosis of T1D was based on the criteria of the International Society for Pediatric and Adolescent Diabetes [15]. The healthy control group was matched with the T1D group by region, sex, age, and time of visit. Cases were excluded if they were < 2 years old, received antibiotic treatment within one month, and presented with infectious diseases, chronic or acute gastrointestinal diseases, other inherited metabolic disorders, and/or serious chronic disorders (Fig. 1). This study was approved by the Ethics Committee of Children’s Hospital of Fudan University ([2019]210), and the guardians of the children provided written informed consent before information and sample collection.

### Sample collection

The clinical characteristics including sex, age, time of visit, fasting blood glucose, HbA1c, and serum C-peptide of T1D patients and healthy control children were obtained through questionnaires administered by the doctors. Fecal samples and first morning urine samples were collected in dedicated sterile containers provided by the research team. Trained medical staff guided and supervised sample collection. Fecal and urine samples were frozen in freezers immediately after collection. Frozen fecal and urine samples were transported on dry ice to the Children’s Hospital of Fudan University within 24 hours of collection and were stored at − 80 °C until analysis.

### Fecal gut microbiota DNA extraction, 16S rRNA sequencing, and bioinformatics analysis

Total bacterial DNA was extracted using a DNA extraction kit according to the manufacturer’s instructions (DNeasy PowerSoil Kit). DNA was diluted to 1 ng/μL and stored at − 20 °C until further processing. The barcoded primers and Takara Ex Taq reagent (Takara, Dalian, China) were used for polymerase chain reaction (PCR) amplification of bacterial 16S rRNA genes. The 16S rRNA V3-V4 region was amplified with universal primers 343F and 798R for bacterial diversity analysis. After purification, the final amplicon was quantified with a Qubit double-strain DNA (dsDNA) assay kit. Equal quantities of purified amplicon were pooled to prepare for subsequent sequencing. After removing low-quality sequences, paired-end reads were assembled using the fast ligation-based automatable solid-phase high-throughput system with a minimum and maximum overlap of 10 bp and 200 bp, respectively, and a 20% maximum mismatch rate [16]. The 16S rRNA sequencing data were analyzed using QIIME software (v.1.8.0), and sequences were assigned to operational taxonomic units using Vsearch software (97% similarity cut-off) [17, 18]. Representative reads were annotated and subjected to a BLAST search against the Silva database (v.123.0) using the Ribosomal Database Project classifier with a 0.70 confidence threshold [19].

### Selection and analysis of urine antibiotics

The selection of antibiotics was mainly based on previous studies [20]. Briefly, 28 antibiotics and five categories of metabolites (macrolide, tetracycline, fluoroquinolone, sulfonamide, and phenicol) were analyzed. All antibiotics were divided into three categories according to use: veterinary antibiotics (VA), human antibiotics (HA), and veterinary/human antibiotics (V/HA). The types of antibiotics that children can use are limited (e.g., fluoroquinolone and tetracycline are restricted for use in children); therefore, most antibiotics in the V/HA group were more likely to be exposed to children through food and water. Thus, VA + V/HA concentrations can reflect exposure to antibiotics in addition to medication. Concentrations of antibiotics were measured by isotope dilution ultra-performance liquid chromatography coupled to quadrupole time-of-flight mass spectrometry (UPLC-Q/TOF MS) according to previously described methods [20].

### Selection and analysis of urine neonicotinoids

Briefly, eight neonicotinoids and four typical metabolites were detected according to previous studies, with isotope dilution UPLC-Q/TOF MS specifically used for neonicotinoid measurement [21].

### Statistical analysis

The concentrations of urine antibiotics and neonicotinoids were adjusted by the urine creatinine levels (Supplementary Table 1). In case of underreporting of the history of antibiotic use in the previous month, we removed data for patients with urine creatinine-adjusted HA concentrations over the 95th percentile. Ultimately, 10 T1D cases and 8 control cases were excluded. Once a measurable value of one kind of antibiotic or neonicotinoid is detected in urine, the child was considered to be exposed to that antibiotic or neonicotinoid. Children were grouped according to the kinds of antibiotics, VA + V/HA or neonicotinoids they were exposed to (no exposure, exposure to one kind, or exposure to two or more kinds). To analyze the combined effect of antibiotics and neonicotinoids exposure, children were also grouped into four groups according to whether they were exposed to one or more kind of antibiotics and/or neonicotinoids (the no ANTI & no NEO group for children without exposure to antibiotics or neonicotinoids; the ANTI & no NEO group for children only exposed to one or more kind of antibiotics; the no ANTI & NEO group for children only exposed to one or more kind of neonicotinoids; and the ANTI & NEO group for children exposed to both one or more kind of antibiotics and one or more kind of neonicotinoids). Furthermore, the association of the urine total concentrations of antibiotics, VA + V/HA or neonicotinoids with clinical characteristics and gut microbiota structure were also analyzed.

Statistical analysis was performed using *R* software (https://www.r-project.org/, version 4.3). For clinical variables, we used *χ*^*2*^ tests to compare categorical variables. Student’s *t* test was used to compare differences between two groups for normally distributed continuous variables, and the Mann–Whitney *U* test was used for variables that were not normally distributed. The indices of α-diversity and β-diversity were calculated in R using the “vegan” and “mixOmics” packages using permutational multivariate analysis, principal component analysis and sparse partial least-squares discriminant analysis. To determine specific taxa, linear discriminant analysis (LDA) effect size was calculated online (http://huttenhower.sph.harvard.edu/galaxy/). A *P* < 0.05 was considered significant.

## Results

### Clinical characteristic of the patients

Among 51 T1D children and 67 healthy control children, 22 (43.1%) and 28 (41.8%) were female, respectively (*P* = 0.883), with a mean age of 7.51 ± 3.61 years and 8.10 ± 2.91 years (*P* = 0.325) (Table 1). The T1D group displayed severely abnormal glucose metabolism, with higher fasting blood glucose levels [17.21 mmol/L (10.55–24.60 mmol/L) vs. 4.90 mmol/L (4.72–5.16 mmol/L)] (*P* < 0.001) and HbA1c levels [11.80% (10.30–13.40%) vs. 5.10% (4.88–5.43%)] (*P* < 0.001) than the control group. The C-peptide concentration of the T1D group was 0.21 μg/mL (0.11–0.40 μg/mL).

**Table 1:** Basic characteristics

Variables	T1D	Control	*P* value
	*n* = 51	*n* = 67	
Female (%)	22 (43.1%)	28 (41.8%)	0.883
Age (y)	7.51 ± 3.61	8.10 ± 2.91	0.325
BMI *z*-score	−1.06 ± 1.57	−0.19 ± 0.94	< 0.001
Fasting blood glucose^a^ (mmol/L)	17.21 (10.55, 24.60)	4.90 (4.72, 5.16)	< 0.001
HbA1c^a^ (%)	11.80 (10.30, 13.40)	5.10 (4.88, 5.43)	< 0.001
C-peptide^a^ (μg/mL)	0.21 (0.11, 0.40)	NA	NA
Urine antibiotics (%)			
All antibiotics (%)	37 (72.5%)	41 (61.2%)	0.197
Human antibiotics (%)	15 (29.4%)	16 (23.9%)	0.499
Veterinary antibiotics (%)	8 (15.7%)	9 (13.4%)	0.730
Veterinary/Human antibiotics (%)	24 (47.1%)	32 (47.8%)	0.940
VA + V/HA (%)	28 (54.9%)	35 (52.2%)	0.774
Macrolides (%)	15 (29.4%)	14 (20.9%)	0.287
Phenicols (%)	4 (7.8%)	6 (9.0%)	0.830
Tetracyclines (%)	5 (9.8%)	7 (10.4%)	0.909
Fluoroquinolones (%)	21 (41.2%)	26 (38.8%)	0.794
Sulfonamides (%)	4 (7.8%)	11 (16.4%)	0.166
All neonicotinoids (%)	36 (70.6%)	35 (52.2%)	0.044

*T1D* type 1 diabetes, *BMI* body mass index, *NA* not available, *VA + V/HA* veterinary and veterinary/human antibiotics. ^a^Data were presented as median (IQR)

### Detection rate of urinary antibiotics and neonicotinoids

Among 28 kinds of antibiotics and metabolites, 18 kinds of antibiotics were found in all 118 urine samples, with an overall detection rate of 66.1% (78/118) (Supplementary Tables 1, 2). The detection rate of all antibiotics in the T1D group was slightly higher than that of the control group, although there was no significant difference (72.5 vs. 61.2%; *P* = 0.197). Among all cases, 43 children (36.4%) were exposed to one kind of antibiotics, while 45 children (38.1%) were exposed to two or more kinds of antibiotics (Supplementary Table 3). The detection rates of HA, VA, V/HA, and VA + V/HA were 26.3% (31/118), 14.4% (17/118), 47.5% (56/118), and 53.4% (63/118), respectively.

Among the five antibiotic categories, fluoroquinolone showed the highest detection frequency at 39.8% (47/118), followed by macrolide (24.6%, 29/118), sulfonamide (12.7%, 15/118), tetracycline (10.2%, 12/118), and phenicol (8.5%, 10/118). The T1D group and the control group showed similar detection rates across categories.

Eleven kinds of neonicotinoids and metabolites were detected in all urine samples [detection rate: 60.2% (71/118)]. There were 37 children (31.4%) exposed to one kind of neonicotinoid, while 34 (28.8%) were exposed to two or more. The neonicotinoid-detection rate in the T1D group was significantly higher than that in the control group (70.6 vs. 52.2%; *P* = 0.044).

### The association between antibiotics/neonicotinoids’ exposure and T1D risks

Children who were exposed to one kind of antibiotics were at higher risk of T1D compared to children without antibiotics exposure [odds ratio (OR) = 2.579, 95% confidence interval (CI): 1.061–6.271; *P* = 0.037] (Supplementary Table 4). Furthermore, children who were exposed to two or more kinds of neonicotinoids also presented higher risk of T1D (OR = 3.911, 95% CI: 1.538–9.945; *P* = 0.004]. When analyzing the combined effect of antibiotics and neonicotinoids, we found that children who were exposed to both one or more kinds of antibiotics and one or more kinds of neonicotinoids had higher risk of T1D than those who were not exposed to antibiotics or neonicotinoids, with the odd ratio of 4.924 (95% CI 1.239–19.572; *P* = 0.024) (Supplementary Table 4).

**Fig. 2:**
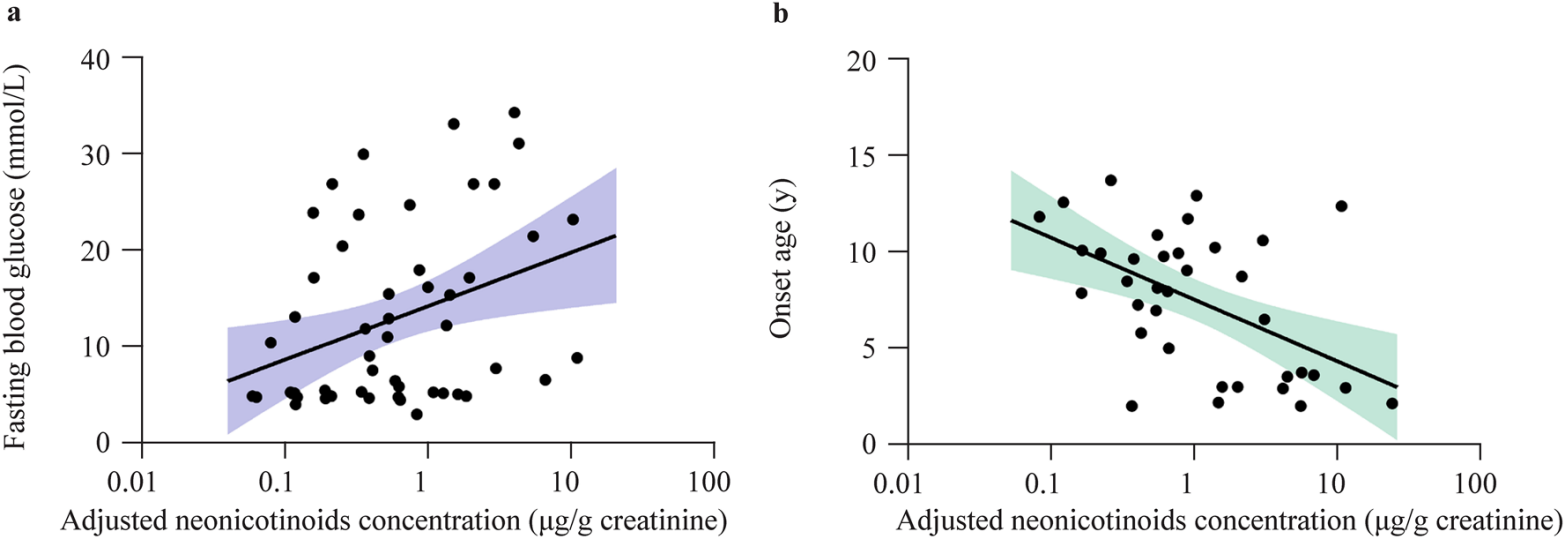
The association between the adjusted neonicotinoids’ concentrations and the fasting blood glucose levels or the onset age of type 1 diabetes (T1D) among T1D children **a** The association between the adjusted neonicotinoids’ concentrations and the fasting blood glucose levels. The violet bar showed the 95% confidence interval (CI); **b** the association between the adjusted neonicotinoids’ concentrations and the onset age of T1D. The green bar showing 95% CI

Moreover, fasting blood glucose levels were elevated according to increases in the urine creatinine-adjusted neonicotinoids concentration (*r* = 0.436, 95% CI 0.720–1.853; *P* < 0.001), with these levels similar only when considering T1D cases (*r *= 0.369, 95% CI 0.201–1.393; *P* = 0.010) (Fig. 2). Among the neonicotinoids-positive T1D cases, the onset age was significantly negatively correlated with the urine creatinine-adjusted neonicotinoids’ concentration (*r* = − 0.398, 95% CI − 0.580 to − 0.063; *P* = 0.026), whereas the urine creatinine-adjusted antibiotics concentration showed no significant relationship with T1D-onset age (*P* = 0.166) or fasting blood glucose (*P* = 0.848).

### Gut microbiota features in T1D

The T1D group showed a characteristic gut–microbiota structure. Both the Shannon index (*P* = 0.016) and Chao1 index (*P* = 0.007) were significantly decreased in the T1D group relative to the control group (Supplementary Fig. 1a, b). Permutational multivariate analysis of variance indicated significant differences in β-diversity between the T1D and control groups (*P* = 0.032, *R*^2^ = 0.022) (Supplementary Fig. 1c).

At the phylum level, the T1D group showed a significantly lower abundance of Firmicutes (*P* = 0.001) and a higher abundance of Proteobacteria (*P* = 0.001) relative to the control group. Furthermore, the T1D group was characterized by a decrease in butyrate-producing genera within Lachnospiraceae and Ruminococcaceae (i.e., *Faecalibacterium*, *Agathobacter*, *Lachnospira*, *Roseburia*, and *Blautia*) and an increase in opportunistic pathogens within Enterobacteriaceae (*Escherichia* and *Shigella*) (Supplementary Fig. 2).

### Association between urinary antibiotic/neonicotinoid concentrations and gut microbiota

#### Alpha diversity and beta diversity

The antibiotics and neonicotinoids’ exposure did not perturb the diversity and richness of gut microbiota. We found no significant difference in the Shannon index and Chao1 index between the control and the T1D group with one kind of, two or more kinds of and without antibiotics, VA + V/HA or neonicotinoids’ exposure (Supplementary Fig. 3). Additionally, β-diversity was similar among these groups. There was also no significant difference of the diversity and richness of gut microbiota among the no ANTI & no NEO group; the ANTI & no NEO group; the no ANTI & NEO group; and the ANTI & NEO group.

#### Differential abundance of taxa

**Fig. 3:**
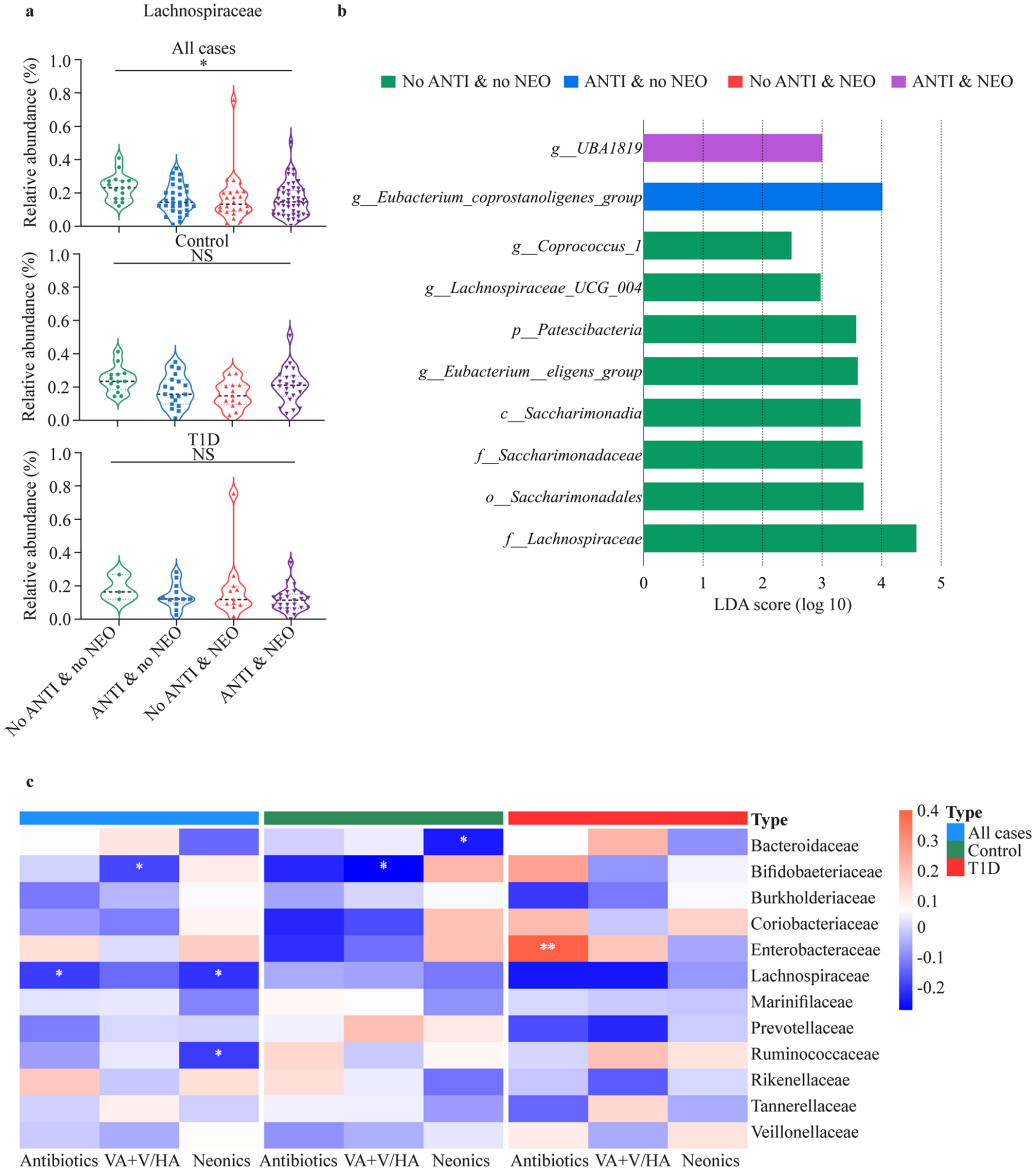
Alterations in gut microbiota between children with different exposure levels to VA + V/HA and neonicotinoids. **a** The relative abundance of Lachnospiraceae among different groups; **b** LDA results among the no ANTI & no NEO group, the ANTI & no NEO group, the no ANTI & NEO group, and the ANTI &NEO group of all cases; **c** The association of adjusted urine VA + V/HA and neonicotinoids’ concentrations and the relative abundance of gut microbiota. The heatmap showed the Spearman’s rank correlation coefficient between the relative abundance of bacterial families with a mean relative abundance ≥ 1% and the adjusted concentration of antibiotics and neonicotinoids. Asterisks represent statistically significant odds ratios. *P < 0.05, †*P* < 0.01. *No ANTI & no NEO* group for children without exposure to antibiotics or neonicotinoids, *ANTI & no NEO* group for children only exposed to one or more kind of antibiotics, *no ANTI & NEO* group for children only exposed to one or more kind of neonicotinoids, *ANTI & NEO* group for children exposed to both one or more kind of antibiotics and one or more kind of neonicotinoids, *LDA* linear discriminant analysis, *VA* + *V/HA* veterinary antibiotics and veterinary/human antibiotics (V/HA), *T1D* type 1 diabetes, *NS* not significant

Separate and combined exposure to antibiotics or neonicotinoids did not significantly affect the structure of gut microbiota both among T1D children and healthy children at the phylum level (Supplementary Fig. 4). However, at the family level, the no ANTI & no NEO group had significantly higher abundance of Lachnospiraceae than the other three groups (*P* = 0.021, Fig. 3a). According to LDA, higher abundance of Lachnospiraceae, including *Eubacterium eligens*, *Coprococcus_1* and *Lachnospiraceae_UCG_004*, and Saccharimonadaceae were found in the no ANTI & no NEO group; while a higher abundance of *Ruminococcaceae_UBA1819* was found in the ANTI & NEO group (Fig. 3b). Furthermore, the abundance of Lachnospiraceae was negatively related with the concentrations of adjusted antibiotics (*r* = −0.20, *P* = 0.031) and neonicotinoids (*r* = −0.21, *P* = 0.022) (Fig. 3c).

## Discussion

This study analyzed the effects of non-therapeutic doses of antibiotics and neonicotinoids in the form of environmental exposure on the gut microbiota of children and their potential association with T1D. The results showed that children exposed to antibiotics and neonicotinoids displayed specific changes in gut microbiota and more serious glucose metabolism disorders.

Because of the inappropriate use of antibiotics and pesticides in animal husbandry, aquaculture, and agriculture, excessive residues of antibiotics and pesticides are found in food, water, soil, dust, and air [22–26]. Children are inevitably exposed to various antibiotics and pesticides in daily life, and the exposure levels were related to living and eating habits [20, 21]. Wang et al. [20] reported an overall detection rate of 56.0% for antibiotics and 50.7% for VA + V/HA in urine from school children in Shanghai. Among preschool and primary school children in Hong Kong of China, the VA detection rate was 77.4% [23]. In the present study, we found slightly higher detection rates for all antibiotics and VA + V/HA compared to that reported by Wang et al. [20], but we found lower than that from the study in Hong Kong, China [23]. The differences in detection rates might be due to various factors, such as diet structure, regional differences, and local restrictions on the use of antibiotics. The detection frequency of neonicotinoids was slightly higher than that for VA + V/HA, with 60.2% found in the present study compared to 81.3% in a previous study among Shanghai children [21]. Although Osaka et al. [27] identified a lower detection frequency of neonicotinoids (58%), they reported detection rates > 90% for other classes of pesticides.

Increasing attention has been focused on the potential health effects associated with intake of undetected antibiotics and neonicotinoids. In addition to antibiotic resistance, long-term exposure to antibiotic residues is related to metabolic disorders, including obesity, type 2 diabetes, immune diseases, and even reproductive problems [8, 9, 28]. Neonicotinoids exert neurotoxic effects on nicotinic acetylcholine receptors and have been associated with disorders related to dysfunctional nervous system development [12]. Additionally, recent studies found that neonicotinoids are related to the development of congenital heart defects and autism spectrum disorders, as well as their associations with immunotoxicity, hepatotoxicity, nephrotoxicity, and reproductive toxicity in mammals [12, 21, 29, 30]. However, most existing studies on the health risks caused by antibiotics and pesticides tend to focus on the effect of high levels of exposure over short periods, because relationships between long-term low-dose exposure and health risks are ambiguous and difficult to study. As a result, the mechanisms associated with their adverse effects on health remain unclear.

The impairment of gut microbiota could represent a direct mechanism by which daily low-dose antibiotic and pesticide exposure affects health; however, a few studies have addressed this. To the best of our knowledge, the present study is the first to systematically analyze the association between the exposure to antibiotics and pesticides and gut microbiota in daily life. We found that high levels of exposure to antibiotics and neonicotinoids did not influence the richness and diversity of gut microbiota. This agreed with a study by Akagawa et al. [31], which indicated that continuous antibiotic prophylaxis of trimethoprim-sulfamethoxazole in children did not alter gut microbiota diversity. However, the findings of the present study did show that exposure to antibiotics and neonicotinoids was associated with small but critical changes to gut microbiota, specifically by perturbing certain taxa and especially butyrate-producing genera, including Lachnospiraceae. Keerthisinghe et al. [32] found that sub-pharmaceutical and dietary exposure levels to tetracycline altered vitamin, nucleotide, and amino acid metabolism by gut microbiota in vitro with a distinct dose–response relationship and induced the release of lipopolysaccharides.

The role of gut microbiota in the onset of immune-related diseases has recently received widespread attention. Reduced bacterial diversity and a decreased abundance of bacteria capable of producing butyrate or lactate were identified in children that later progressed to clinical T1D [33]. The Environmental Determinants of Diabetes in the Young study found that fermentation and biosynthesis of short-chain fatty acids were impaired in T1D children, although this was not consistently associated with particular taxa [34]. In the present study, we found that the abundance of butyrate-producing taxa was consistently lower in children who experienced exposure to antibiotics and neonicotinoids, as well as T1D children. Butyrate exhibits anti-inflammatory effects and helps maintain intestinal-barrier integrity [35]. Thus, our study suggested that exposure to antibiotics and neonicotinoids in daily life might be related to impaired intestinal-barrier function, which might lead to an auto-inflammatory response and autoimmune diseases, including T1D.

This study has some limitations. Different individuals exhibit different responses to the same exposure levels based on their respective gut microbiota. Future research should examine the characteristics of children showing a higher degree of sensitivity to antibiotics and pesticides exposure to define prevention criteria for certain groups. Furthermore, the establishment of birth cohorts to monitor the effects of antibiotics and neonicotinoids’ exposures on gut microbiota, metabolism, and immune levels over a long period of time might help us better understand the relationship between antibiotics and neonicotinoids’ exposures and the onset of T1D.

In summary, this study systematically analyzed the association between daily antibiotics and neonicotinoids’ exposure and the structure of gut microbiota of children with and without T1D. We found that children with exposure to antibiotics and neonicotinoids had small but critical changes in gut microbiota, characterizing by a lower abundance of butyrate-producing genera, especially Lachnospiraceae. Similar changes were also observed in T1D children, which were thought to be associated with the increase of autoimmune level. These findings suggest that exposure to high levels of antibiotics and pesticides in daily life might increase the risk of autoimmune diseases, such as T1D. Future work should focus on relationships between antibiotics and neonicotinoids exposure and the onset of autoimmune diseases in children, as well as the underlying mechanisms.

## Supplementary Information

Below is the link to the electronic supplementary material.

Supplementary file1 (DOCX 1677 KB)

## Data Availability

Data are available on request from the authors.
