# Supplementary material for: Association between new onset type 1 diabetes and real-world antibiotics and neonicotinoids’ exposure-related gut microbiota perturbation
Source: World J Pediatr. 2022 Jul 29;18(10):671–9. doi: 10.1007/s12519-022-00589-3 (PMC9485179; doi:10.1007/s12519-022-00589-3)
Supplement: Supplementary file 1 — Supplementary file1 (DOCX 1677 KB) [file 12519_2022_589_MOESM1_ESM.docx]

**Table 1. Concentrations of antibiotics and neonicotinoid in urine of children**

|  | Percentiles | | | |
| --- | --- | --- | --- | --- |
|  | 50th | 75th | 90th | 95th |
| All antibiotics^†^ | 0.058 | 0.657 | 3.156 | 5.504 |
| Human antibiotics^†^ | 0.000 | 0.015 | 1.137 | 4.158 |
| Veterinary antibiotics^†^ | 0.000 | 0.000 | 0.205 | 0.667 |
| Veterinary/Human antibiotics^†^ | 0.000 | 0.113 | 1.040 | 2.229 |
| VA+V/HA ^†^ | 0.015 | 0.173 | 1.947 | 2.841 |
| All neonicotinoids^†^ | 0.150 | 0.860 | 2.796 | 5.635 |

† urine creatinine-adjusted concentration, μg/g

**Table 2. Concentrations of 28 kinds of antibiotics and 12 kinds of neonicotinoid in urines of children**

|  | P50 | P75 | P80 | P85 | P90 | P95 | P99 | Maximum |
| --- | --- | --- | --- | --- | --- | --- | --- | --- |
| Tetracyclines |  |  |  |  |  |  |  |  |
| Chlorotetracycline^†^ | - | - | - | - | - | - | - | - |
| Tetracycline^†^ | - | - | - | - | - | - | 0.113 | 0.396 |
| Oxytetracycline^†^ | - | - | - | - | - | 0.238 | 2.913 | 10.200 |
| Doxycycline^†^ | - | - | - | - | - | - | 1.851 | 3.602 |
| Fluoroquinolones |  |  |  |  |  |  |  |  |
| Enrofloxacin^†^ | - | - | - | - | - | - | 0.210 | 1.626 |
| Norfloxacin^†^ | - | - | - | - | - | 0.058 | 0.484 | 4.751 |
| Ciprofloxacin^†^ | - | - | 0.024 | 0.063 | 0.122 | 0.611 | 2.182 | 2.571 |
| Ofloxacin^†^ | - | - | - | - | 0.016 | 0.045 | 0.117 | 0.161 |
| Danofloxacin^†^ | - | - | - | - | - | - | - | - |
| Pefloxacin^†^ | - | - | - | - | - | - | - | - |
| Sarafloxacin^†^ | - | - | - | - | - | - | 0.154 | 0.252 |
| Difloxacin^†^ | - | - | - | - | - | - | 0.015 | 0.159 |
| Lomefloxacin^†^ | - | - | - | - | - | - | - | - |
| Macrolides |  |  |  |  |  |  |  |  |
| Azithromycin^†^ | - | - | 0.129 | 0.342 | 0.994 | 3.279 | 16.589 | 19.891 |
| Clarithromycin^†^ | - | - | - | - | - | - | - | - |
| Roxithromycin^†^ | - | - | - | - | - | - | - | - |
| Spiramycin^†^ | - | - | - | - | - | - | - | 0.037 |
| Erythromycin^†^ | - | - | - | - | - | - | 0.691 | 3.793 |
| Sulfonamides |  |  |  |  |  |  |  |  |
| Sulfamethazine^†^ | - | - | - | - | - | - | - | - |
| Acetylated sulfamethazine^†^ | - | - | - | - | - | - | - | 0.126 |
| Sulfadiazine^†^ | - | - | - | - | - | - | - | - |
| Acetylated sulfadiazine^†^ | - | - | - | - | - | - | - | - |
| Sulfamethoxazole^†^ | - | - | - | - | - | - | 0.082 | 0.128 |
| Acetylated sulfamethoxazole^†^ | - | - | - | - | - | - | 0.436 | 2.223 |
| Trimethoprim^†^ | - | - | - | - | - | 0.014 | 0.058 | 0.172 |
| Phenicols |  |  |  |  |  |  |  |  |
| Chloramphenicol^†^ | - | - | - | - | - | - | 0.001 | 0.004 |
| Florfenicol^†^ | - | - | - | - | - | 0.006 | 0.193 | 0.650 |
| Thiamphenicol^†^ | - | - | - | - | - | - | - | - |
| Neonicotinoids |  |  |  |  |  |  |  |  |
| Thiamethoxam^†^ | - | - | - | 0.056 | 0.296 | 0.514 | 2.237 | 7.415 |
| Demethylthiamethoxam^†^ | - | - | - | - | - | - | 0.460 | 1.279 |
| Clothianidin^†^ | - | - | - | - | 0.258 | 0.455 | 1.235 | 1.583 |
| Demethylclothianide^†^ | - | - | - | 0.242 | 0.372 | 0.708 | 3.647 | 10.585 |
| Imidacloprid^†^ | - | - | - | - | - | - | 0.100 | 0.182 |
| Imidacloprid hydroxylated^†^ | - | - | - | - | - | - | - | 1.842 |
| Acetamiprid^†^ | - | - | - | - | - | - | - | - |
| Demethylacetamiprid^†^ | - | 0.346 | 0.416 | 0.540 | 1.049 | 1.567 | 2.559 | 9.871 |
| Thiacloprid^†^ | - | - | - | - | - | - | - | - |
| Nitenpyram^†^ | - | - | - | - | - | 0.665 | 5.467 | 11.725 |
| Imidaclothiz^†^ | - | - | - | - | - | - | - | - |
| Dinotefuran^†^ | - | - | - | - | - | - | - | 0.665 |

† urine creatinine-adjusted concentration, μg/g

**Table 3.** Kinds of antibiotics and neonicotinoids children were exposed to

|  | T1D | Control |
| --- | --- | --- |
| Antibiotics |  | 0.176 |
| 0 | 14 (27.5) | 26 (38.8) |
| 1 | 25 (49.0) | 18 (26.9) |
| 2 | 6 (11.8) | 15 (22.4) |
| 3 | 5 (9.8) | 6 (9.0) |
| ≥4 | 1 (2.0) | 2 (3.0) |
| Neonicotinoids |  | 0.011 |
| 0 | 15 (29.4) | 32 (47.8) |
| 1 | 14 (27.5) | 23 (34.3) |
| 2 | 11 (21.6) | 6 (9.0) |
| ≥3 | 11 (21.6) | 6 (9.0) |

**Table 4. The association of antibiotics/neonicotinoids exposure and T1D the binary logistical regression model**

|  | OR | 95% CI | *p* value |
| --- | --- | --- | --- |
| Groups based on the kinds of antibiotics | | | |
| 0 | Reference |  |  |
| 1 | 2.579 | 1.061-6.271 | 0.037 |
| ≥2 | 0.969 | 0.373-2.514 | 0.948 |
| Groups based on the kinds of neonicotinoids | | | |
| 0 | Reference |  |  |
| 1 | 1.299 | 0.526-3.207 | 0.571 |
| ≥2 | 3.911 | 1.538-9.945 | 0.004 |
| Groups based on the exposure to antibiotics and neonicotinoids | | | |
| no ANTI & no NEO | Reference |  |  |
| ANTI & no NEO | 2.737 | 0.643-11.651 | 0.173 |
| no ANTI & NEO | 3.667 | 0.826-16.273 | 0.087 |
| ANTI & NEO | 4.924 | 1.239-19.572 | 0.024 |

*ANTI* antibiotics; *NEO* neonicotinoids; *OR* odd ratio; *CI* confidence interval.


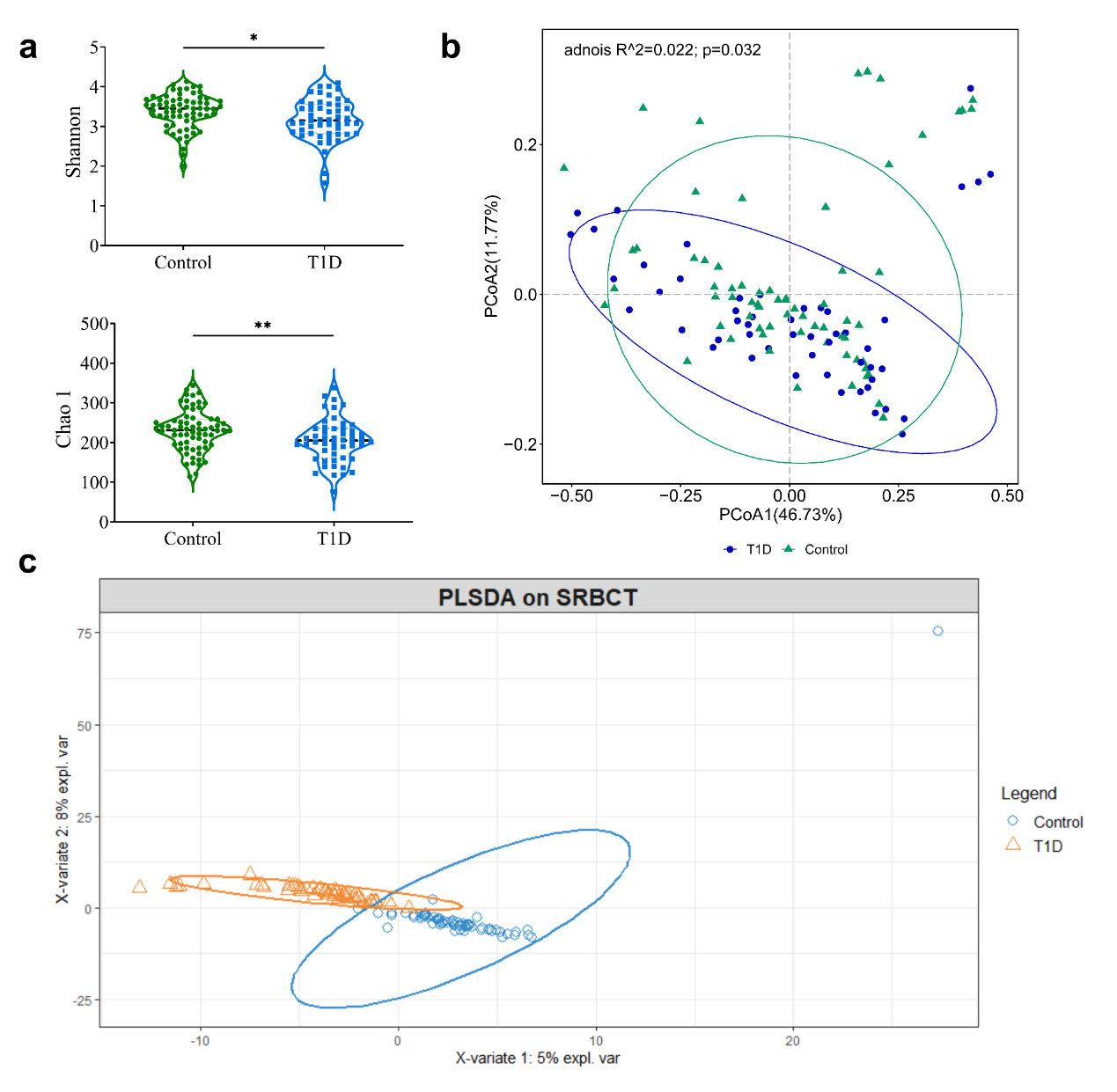


**Fig. 1** Changes in gut microbiota in T1D

(a) Diversity of microbial communities (Shannon index) and richness of microbial communities (Chao1 index). (b) Principal component analysis based on weighted UniFrac distance. (c) sPLS-DA graph. *T1D* type 1 diabetes, * *P* < 0.05, ***P* < 0.01


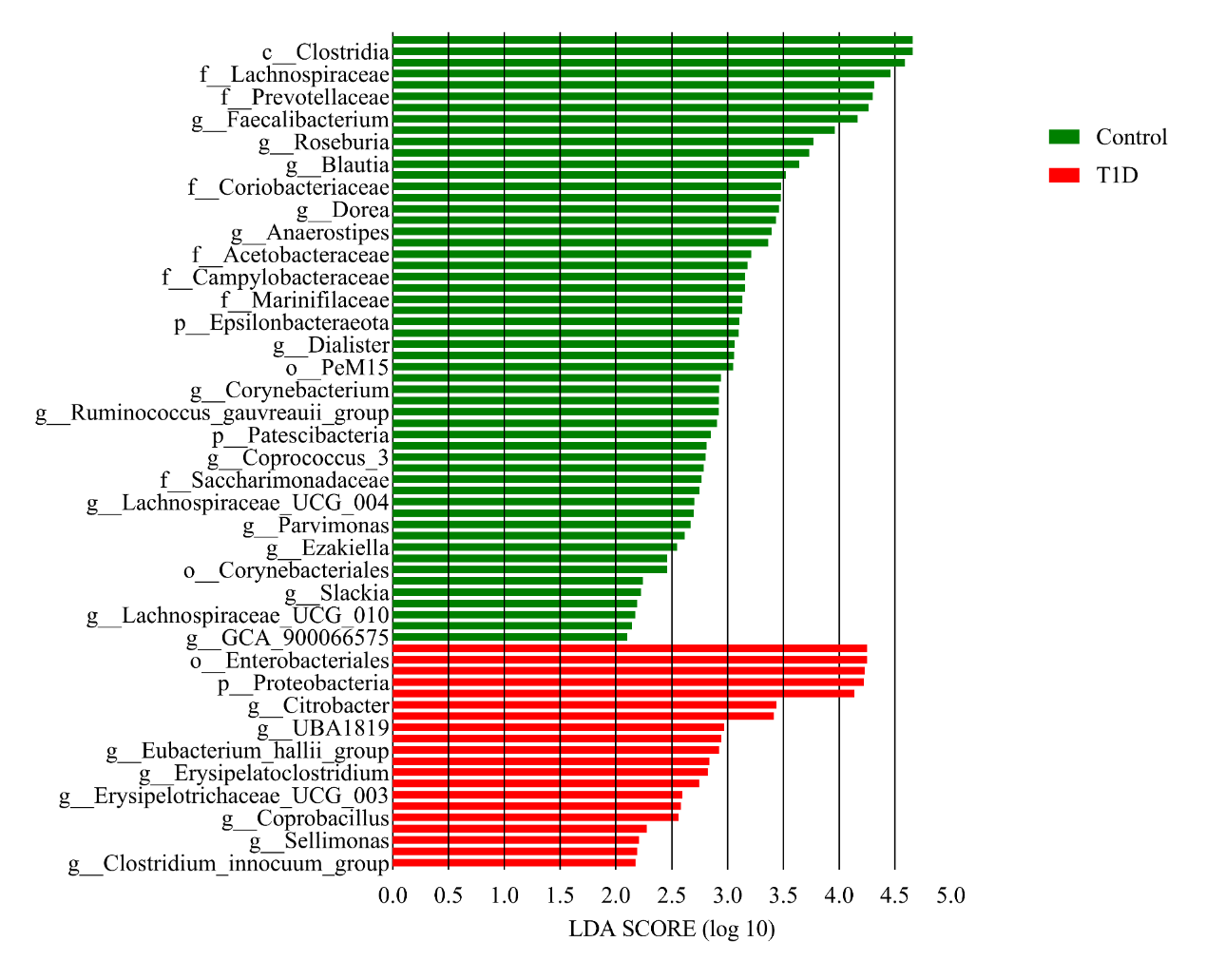


**Fig. 2** Alterations in gut microbiota between children with T1D and control children


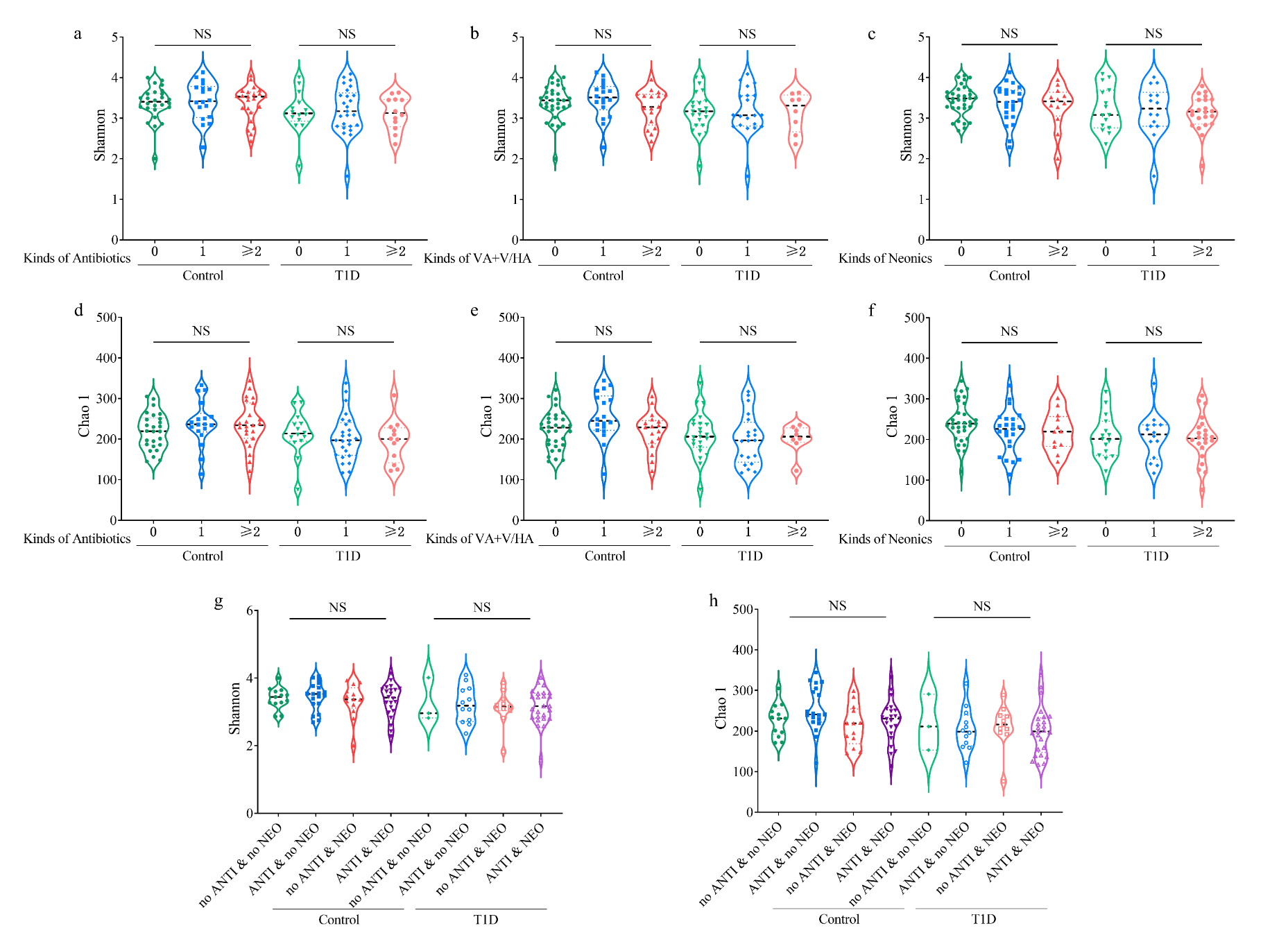


**Fig. 3** Changes of alpha diversity in the T1D and control group respectively

(a, b, c, g) Diversity of microbial communities (Shannon index). (d, e, f, h) Richness of microbial communities (Chao1 index). *T1D* type 1 diabetes, *NS* no significant, *No ANTI & no NEO* group for children without exposure to antibiotics or neonicotinoids, *ANTI & no NEO* group for children only exposed to one or more kind of antibiotics, *no ANTI & NEO* group for children only exposed to one or more kind of neonicotinoids, *ANTI & NEO* group for children exposed to both one or more kind of antibiotics and one or more kind of neonicotinoids


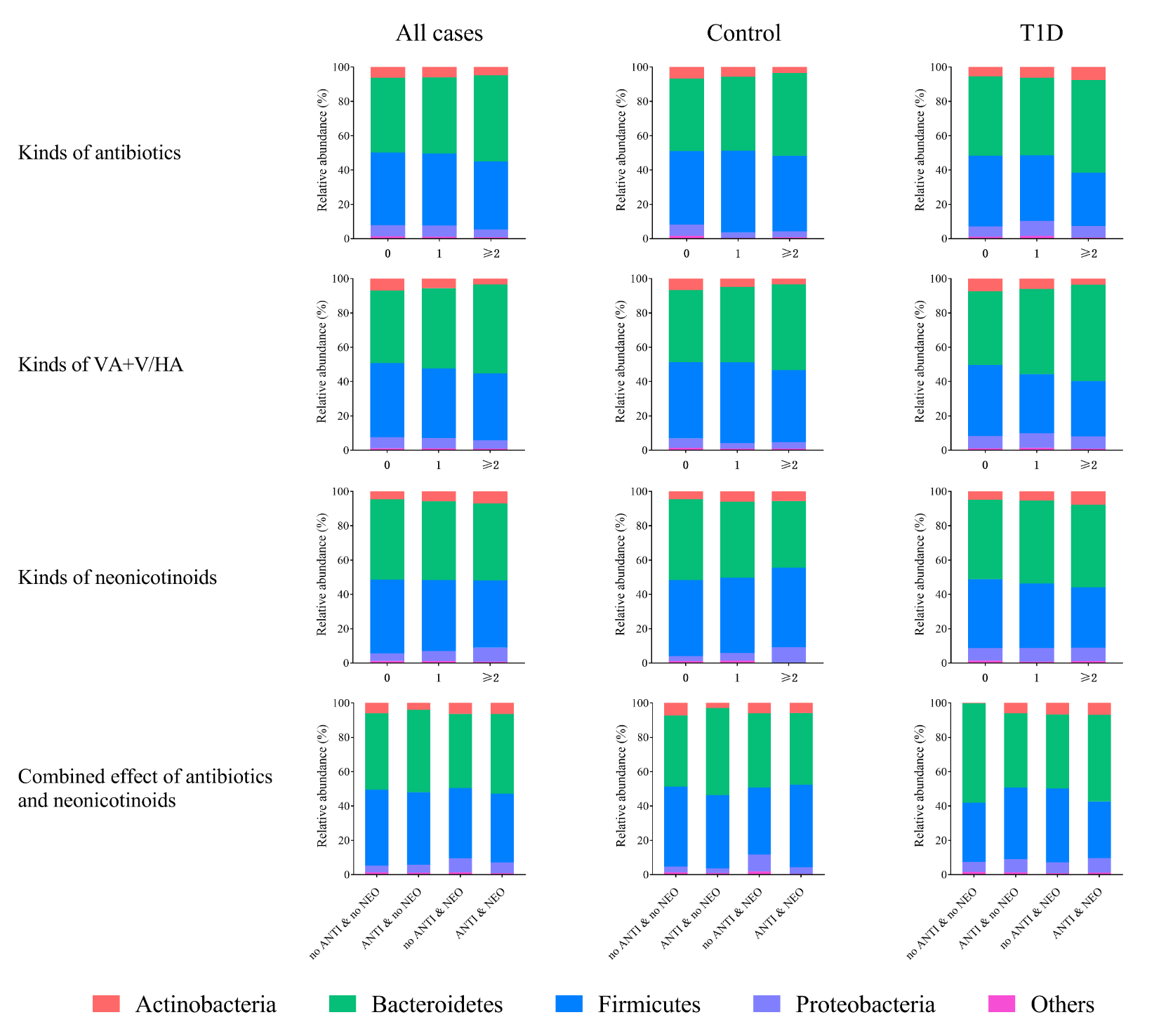


**Fig. 4** Changes in the relative abundance of gut microbiota at the phylum level between children with and without antibiotics, VA+V/HA and neonicotinoids exposures among the control group and the T1D group

Taxa with a relative abundance <1% in all samples were merged with others.
